# Impact of DHA on Metabolic Diseases from Womb to Tomb

**DOI:** 10.3390/md12126190

**Published:** 2014-12-18

**Authors:** Ilse A. C. Arnoldussen, Amanda J. Kiliaan

**Affiliations:** Department of Anatomy, Donders Institute for Brain, Cognition & Behaviour, Radboud University Medical Center, Geert Grooteplein Noord 21, 6525 EZ Nijmegen, The Netherlands; E-Mail: Ilse.Arnoldussen@radboudumc.nl

**Keywords:** docosahexaenoic acid, humans, metabolic diseases, cardiovascular disease, obesity, diabetes mellitus type II

## Abstract

Long chain polyunsaturated fatty acids (LC-PUFAs) are important mediators in improving and maintaining human health over the total lifespan. One topic we especially focus on in this review is omega-3 LC-PUFA docosahexaenoic acid (DHA). Adequate DHA levels are essential during neurodevelopment and, in addition, beneficial in cognitive processes throughout life. We review the impact of DHA on societal relevant metabolic diseases such as cardiovascular diseases, obesity, and diabetes mellitus type 2 (T2DM). All of these are risk factors for cognitive decline and dementia in later life. DHA supplementation is associated with a reduced incidence of both stroke and atherosclerosis, lower bodyweight and decreased T2DM prevalence. These findings are discussed in the light of different stages in the human life cycle: childhood, adolescence, adulthood and in later life. From this review, it can be concluded that DHA supplementation is able to inhibit pathologies like obesity and cardiovascular disease. DHA could be a dietary protector against these metabolic diseases during a person’s entire lifespan. However, supplementation of DHA in combination with other dietary factors is also effective. The efficacy of DHA depends on its dose as well as on the duration of supplementation, sex, and age.

## 1. Introduction

Since the early 1970s, scientists have studied the role of omega-3 LC-PUFA docosahexaenoic acid (DHA) in human health. Currently, it has been shown that DHA may be beneficial in several human pathologies like cardiovascular diseases, obesity, and diabetes mellitus type 2 (T2DM); DHA is associated with a reduced incidence of stroke, atherosclerosis, lower bodyweight and decreased T2DM prevalence [1,2,3,4,5,6,7]. Nevertheless, research findings are ambiguous and argue over the supplemented dose and duration of the supplementation in relation to sex and age differences. In this review, the impact of long chain polyunsaturated fatty acids (LC-PUFAs) on current societally highly relevant metabolic diseases in humans will be discussed, and, in particular, the role of DHA in connection with diabetes type II (T2DM), obesity, and cardiovascular disease (CVD). The following categories are described in this review: childhood, adolescence, adulthood, as well as mature and late adulthood, to indicate the effect of DHA on metabolic diseases in critical stages of life.

We searched the PubMed database for original and review articles in English, published from 1993 to August 2014. The main search topics concerned DHA, the influence of DHA on disorders such as T2DM, obesity and CVD. The search strategy was based on the following search terms: DHA, LCPUFA, obesity, T2DM, CVD, childhood, adolescence, adulthood, and the elderly. Moreover, we filtered our total list of relevant papers by hand, in order to identify new, potentially relevant papers. We selected the studies based on titles and abstracts, a selection of these two components was not sufficient, we evaluated the whole publication instead.

### Docosahexaenoic Acid (DHA)

**Figure 1:**
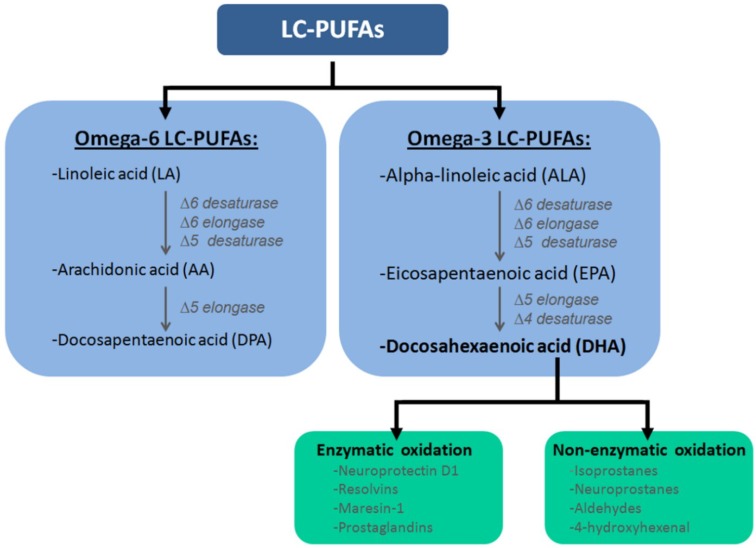
Classification of long chain polyunsaturated fatty acids (LC-PUFAs). PUFAs are subdivided into two subclasses: omega-6 and omega-3 PUFAs. Within the omega-6 PUFAs class precursor fatty acid, (*i.e.*,) linoleic acid (LA), is converted into arachidonicacid (AA) via ∆6-desaturase, ∆6-elongase and ∆5-desaturase. Subsequently, AA can be metabolized into docosapentaenoic acid (DPA) via ∆5-elongase. In the omega-3 fatty acid class, the precursor fatty acid,* i.e.*, α-linoleic acid (ALA), is converted into eicosapentaenoic acid (EPA) via ∆6-desaturase, ∆6-elongase and ∆5-desaturase, which can subsequently be converted into docosahexaenoic acid (DHA) via ∆5-elongase and ∆4-desaturase. DHA is the precursor of several bioactive products, because it can be converted via enzymatic and non-enzymatic oxidation. DHA can be metabolized into e.g., resolvins E1 & E2 and D1–D5, maresin-1 and neuroprotectin D1 by means of enzymatic oxidation. These eicosanoids can enter the nucleus where they downregulate gene expression, promote inflammation or enter the bloodstream. This is where they reduce platelet aggregation and accumulation of fatty acids in the arteries. In non-enzymatic oxidation, DHA can be oxidized by radical oxygen species into lipid peroxides such as neuroprostanes, isoprostanes, aldehydes and 4-hydroxyhexenal. These peroxides can activate transcription factors such as peroxisome proliferator-activated receptors (PPARs) and nuclear factor-like 2 (Nrf2), which upregulate gene expression in the nucleus. (∆6/∆5/∆4: at the double bond at the 6th/5th/4th position).

Humans need to obtain adequate levels of essential fatty acids from dietary sources. These lipids are important constituents of phospholipids, which are the building blocks of all membranes. An important constituent is the essential fatty acid alpha-linolenic acid (ALA), which can be found, for example, in soy beans, flaxseed, linseed and walnut oils [8]. Based on its molecular structure, three double *cis*-bonds and an 18-carbon chain, ALA is classified as a long chain polyunsaturated fatty acid (LC-PUFA) (Figure 1). ALA can be converted into two other LC-PUFAs: docosahexaenoic acid (DHA) and eicosapentaenoic acid. The first step in this process is to convert ALA into an active metabolic product,* i.e.*, eicosapentaenoic acid (EPA) (Figure 1) [9,10]. This is done through a double bond creation at the 6th and 5th position (∆6- and ∆5-desaturase), and an elongase at the double bond at the 6th position (∆6-elongase). Subsequently, EPA can be metabolized into DHA via ∆5-elongation and ∆4-desaturation (Figure 1) [9,10]. The described process is limited, and it only occurs in the liver, the cerebrovascular lumen, and astroglial cells [9,11,12]. DHA can be converted into potent novel molecules with anti-inflammatory and organ-protective properties [13,14,15,16,17] such as the specialized pro-resolving lipid mediators (SPMs), including D- and E-series resolvins, neuroprotectins, and maresins (Figure 1) [15,16,17,18,19] via specialized chemical mediators.

DHA can also be obtained directly from dietary sources such as deep-sea fish, whereby white fish such as cod and haddock contain a lower concentration of DHA than oily fish like Atlantic salmon, blue mackerel and sardines [8,20,21]. In cold-water deep sea fish, DHA is accumulated through consumption of marine microalgae which are able to synthesize DHA [22]. Cold-water deep-sea fish that live in oceanic seas with high water pressure and low temperatures need adequate levels of long chain poly-unsaturated fatty acids like DHA to maintain functional membrane fluidity. Neuronal membranes containing high concentrations of saturated fatty acids would become rigid and lose functionality in these cold oceanic seas [23].

DHA is an essential component in phospholipids because it maintains membrane fluidity. Membrane fluidity can be defined as the optimum transition point between gel and liquid crystal where the neuronal lipid bilayer can exist. This condition is of physiological importance for signal transmission. It can be strongly influenced by fatty acid composition and should therefore be maintained optimal [24]. Dietary intake of specific fatty acids can modify the lipid composition of neurons up to a certain degree [24]. Prolonged intake of DHA, for example, will modestly increase brain DHA content and decrease brain omega-6 PUFAs content simultaneously, particularly docosapentaenoic acid (DPA) [25]. High concentrations of DHA within the lipid bilayer provide neuronal membranes with the flexibility/fluidity that is required in order to function properly during axonal and synaptic growth, and improve functioning of ion-channels and receptors through better transmission [14,24,26,27,28]. This is contrary to the fact that complete long term dietary deprivation of omega-3 PUFAs depletes the brain of DHA, but will specifically increase the brain DPA content of the omega-6 PUFAs family [24]. Thus, the concentration of unsaturated fatty acids will be more or less maintained, but the functionality of the membranes can differ drastically because of changes in omega-3 and omega-6 ratios. In addition, membrane fluidity will change, possibly causing impairment of proper membrane function and signal transmission [24].

It has been demonstrated that in mammals, DHA, in free form, rapidly crosses the blood brain barrier (BBB), similar to freely diffusible lipophilic drugs [29]. The first transport mechanisms of omega-3 PUFAs into the cell is described as a rapid diffusion flip-flop across cell membranes [30]. Others suggest a protein mediated uptake via fatty acid transport proteins (FATPs), fatty acid binding proteins (FABPs), fatty acid translocases (FATs), long chain fatty acyl-CoA synthetases (ACSL) or long chain fatty acyl-CoA binding proteins (ACBPs) [31,32,33,34,35,36,37,38]. Flip-Flop diffusion and protein-mediated transmembrane transfer seem to co-exist as two separate ways of fatty acid transport into the cell, in which the nature of the membrane, type and functional state of the cell, availability and type of fatty acids and differences in hormonal environment could be important [39,40].

## 2. Metabolic Diseases

There is an emerging epidemic of obesity in people among all ages in Western societies, and it is now burgeoning in non-Western societies. Each year, obesity or obesity-related health conditions lead to the death of 2.8 million adults around the world [41]. Even though it has an undefined aetiology, it is generally caused by an imbalance of energy intake* versus* energy output. This results in excessive adipose tissue accumulation that may eventually have an adverse effect on human health. The epidemic of overweight and obesity has caused a dramatic increase in the number of individuals who are suffering from T2DM and premature cardiovascular disease (pCVD) [42]. In the U.S., for example, two-thirds (167 million) of adults are diagnosed as being overweight or obese, and another 26 million have T2DM [43]. It has been estimated that approximately 80% of all new T2DM cases are due to obesity [44]. Currently, T2DM is the most common metabolic disorder, and it has reached epidemic proportions in many countries [45]. Insulin resistance and inflammation have played a central role in the pathogenesis of T2DM and both have developed long before the onset of the disease [45]. The two to fourfold increase in cardiovascular morbidity as well as mortality in diabetic individuals is a major concern [45]. T2DM increases the risk of both heart disease and stroke as 50% of people with diabetes die from CVD [46]. Insulin signaling plays a critical role in normal vascular function via endothelial cell nitric oxide production, modulation of calcium handling and sensitivity in vascular smooth muscle cells [43].

Patients with T2DM and/or obesity have a significantly increased risk of heart attack and stroke when compared to people who have a normal insulin sensitivity and a normal weight [43]. Thus we can conclude that metabolic diseases like CVD, T2DM and obesity, are highly intercorrelated and reinforce each other. Studies have shown that DNA, as a dietary component, has beneficial effects on these three metabolic diseases [2,3,6,47,48,49,50]. DHA supplementation seems to affect symptoms of metabolic diseases by reducing plasma triglyceride levels, increasing glucose and insulin sensitivity, as well as reducing atherosclerosis. DHA could affect these characteristics through mediating processes such as reducing adipocyte differentiation, decreasing adipocyte apoptosis, improving lipolysis as well as insulin metabolism, decreasing production of proinflammatory cytokines, improving endothelial function and activating large-conductance calcium and voltage-activated potassium (BK) channels [51,52,53,54,55].

### 2.1. Processes Mediated by DHA

#### 2.1.1. Inflammation

It has been shown that DHA is capable of reducing several inflammatory mechanisms which can be of importance in the pathology of CVD, T2DM and obesity. For instance, in Pickup* et al.* [56] it was hypothesized that the innate immune system is involved in the pathogenesis of T2DM. Tumor necrosis factor alpha (TNF-α), as well as interleukin-1 & 6 (IL-1 & IL-6) are known to play a major role in the pathophysiology of insulin resistance in T2DM [57]. Moreover, endothelial dysfunction in cardiovascular disease involves many inflammatory processes, and, for instance cytokine overproduction is associated with endothelial dysfunction and atherosclerosis [51,52]. For example, TNF-α can accelerate experimental atherosclerosis processes* in vivo* in human and rat veins, mainly through induction of adhesion molecule expression in endothelial and vascular smooth muscle cells, resulting in altered endothelium-dependent vasodilation and promotion of endothelial cell apoptosis [51,52,58,59]. In obesity, low state chronic adipose inflammation is an early characteristic and DHA is capable of decreasing this low state chronic adipose inflammation [60]. For example, DHA is able to downregulate tumor necrosis factor-α (TNF-α), interleukin-6 (IL-6) and monocyte chemotactic protein-1 (MCP-1) secretion in human adipose tissue and adipocytes cultures [61]. Resolvin D1, an anti-inflammatory SPM converted from DHA (Figure 1), increases both the number of macrophages containing ingested particles and the number of phagocytosed particles in adipose tissue, and also reduces macrophage reactive oxygen species production [62]. In conclusion, these findings illustrate novel mechanisms through which resolvin D1 and its precursor DHA may confer anti-inflammatory actions [62].

#### 2.1.2. Insulin Metabolism

DHA may be able to partly restore insulin resistance, which is associated with an increased risk of developing T2DM [63,64,65,66,67]. In an obesity mouse model (ob/ob), dietary intake of omega-3 PUFAs induced an increase in adiponectin, a regulator to maintain the energy balance [53]. Bioactive SPMs, resolvins E1 and NPD1 (Figure 1), mimicked the insulin-sensitizing effects and induced adiponectin expression to a similar extent to that of rosiglitazone, a member of the thiazolidinedione family of antidiabetic drugs [53]. Together, these findings reveal effects of DHA and its bioactive SPMs in preventing obesity-induced insulin resistance via mimicking insulin sensitizing and expression of adiponectin.

#### 2.1.3. Adipocytes

It has been demonstrated that DHA is involved in reducing symptoms of obesity via inhibiting adipocyte differentiation [60,67,68,69,70,71,72,73]. The direct effect of DHA on cell growth, differentiation, apoptosis, and lipolysis in adipocyte cell cultures was investigated [74]. Proliferation of preconfluent pre-adipocytes was not affected by the DHA treatment, but by DHA inhibited differentiation-associated mitotic clonal expansion [74]. DHA decreased mean droplet size and percent lipid area in adipocytes in a dose-dependent manner and, moreover, postconfluent pre-adipocytes demonstrated apoptosis after DHA treatment [74]. Additionally, DHA increased basal lipolysis in fully differentiated adipocytes [74]. These findings indicate that DHA may exert its obesity lowering effect by inhibiting differentiation of pre-adipocytes, inducing apoptosis in postconfluent pre-adipocytes and promoting lipolysis in adipocyte cell culture [74].

#### 2.1.4. Cardiovascular Health

It is well-known that omega-3 LC-PUFAs are able to improve vascular function; these effects were first described in Greenland Inuits [75]. Research revealed that omega-3 LC-PUFAs prevent myocardial infarction and arrhythmia, decrease systolic and diastolic blood pressure and improve vascular function [76,77,78,79,80]. Individuals receiving omega-3 LC-PUFA supplementation, for example, had a decreased risk ratio in cardiac events like atrial fibrillation, nonfatal myocardial infarction and stroke [76,78,79,80]. Omega-3 LC-PUFAs have been shown to decrease systolic and diastolic blood pressure and development of hypertension [77]. In Thies* et al.* it was reported that consumption of omega-3 LC-PUFAs contributed to the stability of atherosclerotic plaques [81]. In Baumann* et al.* it was shown that the transcription of pro-inflammatory molecules was decreased after taking an omega-3 LC-PUFA supplementation for four weeks, followed by an improved vascular reactivity and endothelial function [80,82]. In addition, omega-3 LC-PUFAs can improve vascular function via endothelium relaxation [83]. It has been shown in* in vivo* studies that endothelium relaxation was higher in rodents and humans treated with omega-3 PUFAs [58,59,84]. Moreover, it was reported that DHA rapidly and reversibly activates BK-channels [54]. In vascular smooth muscle cells, BK-channels provide a critical vasodilatory influence [54]. BK channels are thus possible receptors for DHA and by activating these BK channels, blood pressure may be lowered [54].

### 2.2. DHA & Metabolic Diseases in Childhood

Prevalence of childhood overweight and obesity has rapidly increased worldwide, and at the same time prevalence of T2DM and CVD has increased, even from a very early age [85]. To illustrate this, two-thirds of severely obese children aged 12 or younger develop cardiovascular risk factors [85]. In a study of Salvatore* et al.* [86] performed in the USA, 80% of the obese children had a low high-density lipoprotein (HDL) cholesterol level which is a risk factor for atherosclerosis [86]. In the Netherlands in 2009, 13.3% of the boys and 14.9% of the girls were diagnosed with obesity, and 62% of these obese children aged 12 or younger developed hypertension [85]. It was shown that a daily supplementation of omega-3 LC PUFA improves vascular function and lowers the degree of inflammation in obese adolescents [47]. Interestingly, daily consumption of 300 mg DHA and 42 mg for a period of three weeks led to improvement of the anthropometric and lipid parameters in obese children aged 8–12 years [87]. Damsgaard* et al.* found that DHA plasma levels were positively associated with mean arterial pressure in boys. Moreover, girls had a higher body fat percentage, increased diastolic blood pressure and heart rate, elevated plasma triglyceride and insulin levels, and increased insulin resistance and glycosylated hemoglobin in comparison to boys [88]. Burrows* et al.* [2] and Decsi* et al.* [3] revealed that lower levels of DHA in plasma phospholipids are associated with insulin resistance, T2DM and obesity in children. Moreover, Juarez-Lopez* et al.* found that a LC-PUFA supplementation for a 12-week period reduced BMI and decreased the concentrations of glucose, insulin, triglyceride-levels in the plasma of obese children [89]. These findings indicate that an adequate level of DHA may be of importance in the prevention of these chronic diseases in later life [2,3].

Thus, DHA levels in childhood are associated with reduced plasma insulin, glucose and triglyceride levels. These are all characteristics of obesity and T2DM, and, therefore, an adequate LC-PUFA level may be of great importance in preventing metabolic diseases later in life. Nonetheless, other LC-PUFAs such as EPA may contribute towards this prevention as well as factors such as sex and age differences.

### 2.3. DHA & Metabolic Diseases in Adolescence

Obesity in children continues to evolve into adolescence, and thereby it contributes to the risk of health problems like insulin resistance, hypertension and even brain abnormalities [85,90,91]. Omega-3 PUFAs supplementation , including DHA, has beneficially affected female obesity in adolescence as it improved glucose tolerance by 39% and restored insulin concentration by 34% during an intravenous glucose tolerance test (IVGTT) [92]. In contrast, with regard to male obesity in adolescence, neither of these two parameters were influenced by omega-3 PUFAs supplementation [92]. In conclusion, omega-3 PUFAs supplementation seems to be beneficial in restoring insulin concentration and glucose tolerance in adolescent girls [92], which additionally indicates that sex differences can be differentially affected by omega-3 LC-PUFA supplementation (Table 1).

Risé *et al.* examined blood fatty acid levels particularly LC-PUFA levels in Italian neonates, children, adults and the elderly, and they found that LC-PUFA levels indicated significant differences due to age and sex [93]. In addition, Damsgaard *et al.* and Dangardt* et al.* found differences in DHA plasma levels, glucose tolerance, diastolic blood pressure, heart rate, plasma triglyceride and insulin levels, homeostasis model assesment-insulin resistance and glycosylated levels between sexes [88,92]. These findings emphasize that the differing need for LC-PUFAs like DHA depends on age and sex. Future research should therefore focus on these diverse needs of sex and age.

### 2.4. DHA and Metabolic Diseases in Adults

Prevalence of obesity, T2DM and CVD increases enormously in adults in the western world [41,94]. In the US in 2012, 167 million adults were obese or overweight, 36 million adults had CVD and 24 million adults suffered from T2DM [95]. Labonte* et al.* [96] suggested a minor impact of DHA on T2DM incidence in adults [97]. However, Stirban *et al.* [98] revealed that individuals with T2DM who received an omega-3 LCPUFA supplementation for a period of six weeks showed a postprandial decrease in macrovascular function (Table 1) [98]. These observations suggest that omega-3 LC PUFAs protect the macrovascular function in individuals with T2DM [98].Secondly, Virtanen and colleagues reported that men with higher serum level of LC-PUFAs had a 33% lower multivariate-adjusted risk for T2DM [99]. (Table 1). Purified DHA supplementation reduced both collagen aggregation and collagen-stimulated thromboxane release in hypertensive diabetic individuals, making DHA an effective anti-thrombotic agent [100]. In hypertensive T2DM patients it was shown that a DHA and EPA supplementation during eight weeks can diminish platelet superoxide production, which suggests a therapeutic role for DHA as well as EPA in reducing vascular-derived oxidative stress associated with T2DM [101]. Supplementation with purified DHA reduces serum triglycerides and collagen aggregation, increases high-density lipoprotein 2 (HDL2) cholesterol, besides improving blood pressure [100,102,103,104,105]. In obese adults, higher plasma levels of total omega-3 PUFAs are associated with a healthier BMI, waist circumference and hip circumference [6]. DHA supplementation decreased plasma triglycerides and VLDL-triglycerides in noninsulin-dependent diabetes mellitus (NIDDM) hypertriglyceridemia patients and hypertriglyceridemic men [106,107]. Moreover, supplementation with conjugated linoleic acids (CLAs) plus omega-3 LC-PUFAs prevents increased abdominal fat mass and raises fat-free mass and adiponectin levels in obese adults, without deleteriously affecting insulin sensitivity, whereas in lean adults these parameters were unaffected [108]. More specifically, LC-PUFA supplementation decreased the gene expression of most analyzed inflammatory genes in subcutaneous adipose tissue, and increased production of anti-inflammatory eicosanoids in visceral adipose tissue and subcutaneous adipose tissue [109]. Furthermore, it has been shown that treatment with omega-3 PUFAs diminishes adipocyte differentiation, stimulates adipocyte apoptosis, improves lipid metabolism and systemic inflammation [5,62,84,98,108,109,110], through previously described processes such as anti-inflammatory, adipocyte apoptosis and differentiation.

A well-studied effect of LC-PUFAs, EPA and DHA is that intake of these fatty acids protects against cardiovascular disease because it decreases blood pressure and atherosclerosis formation [75,111,112,113,114]. These beneficial effects are due to the capacity to prevent arrhythmias, thus improving vasoactivity, decreasing blood pressure and inflammation and decreasing atherosclerosis [75,83,110,111,112,113,114,115,116,117,118,119,120]. The advantages of taking omega-3 LC-PUFA supplementation are that arrhythmias is prevented, vasoactivity is improved and blood pressure, inflammation and atherosclerosis are decreased [1,75,83,110,111,112,113,114,115,116,117,118,119,120,121]. Nevertheless, it was revealed that in healthy adults DHA supplementation did not improve the endothelial function [7].

Thus, omega-3 LC-PUFA supplementation may mainly affect obesity in adults by reducing adiposity and inflammation, and LC-PUFAs may improve vascular function by reducing the blood pressure, heartrate and coronary calcification. Nonetheless, research findings are ambiguous and therefore more research is needed, while taking dosing and duration of LC-PUFA or DHA supplementation into account.

**Table 1:** DHA and metabolic diseases through lifetime.

Lifetime	Author	Year	N	Age (Years)	DHA	Outcome
**Childhood**						
	Desci [3]	2002	80 ♂ & ♀	12	Plasma phospholipids ARA & DHA	Values of arachidonic acid and docosahexaenoic acid were significantly lower in diabetic children than in controls.
	Burrows [2]	2011	48 ♂ & ♀	Non-obese: 9.0 ± 0.9 Obese: 8.9 ± 1.2	Erythrocyte fatty acid; the Omega-3 index (O3I) composition	Obese children had altered erythrocyte fatty acid composition unrelated to reported dietary intake. A greater proportion of obese children had an omega-3 index of <4.0 (high risk) compared with non-obese children.
	Vasickova [87]	2011	120 ♂ & ♀ (obese)	10.0 ± 1.9	300 mg/day DHA + 42 mg/d EPA for 3 weeks	Daily consumption of 300mg DHA and 42 mg EPA for three weeks leads to an improvement of the anthropometric and lipid parameters in obese children [87].
	Juarez Lopez [89]	2013	201 ♂ & ♀ (obese and insulin resistant)	11.6 ± 0.7	12 weeks LC-PUFA supplementation, 360 mg EPA & 240 mg DHA daily	LC-PUFA supplementation for 12 weeks decreased the concentrations of glucose, insulin, triglyceride-levels and BMI.
	Damsgaardt [88]	2013	73 ♂ & ♀	10.29 ± 0.58	Plasma DHA &EPA concentrations	DHA was positively associated with mean arterial pressure in boys.
**Adolescence**						
	Dangardt [92]	2012	25 ♂ & ♀	15.6 ± 0.9 ♀ 15.7 ± 1.0 ♂	1,2 g/d LC-PUFAs (DHA & EPA) for 3 months	Three months of supplementation of omega-3 LCPUFA improved glucose and insulin homeostasis in obese girls without influencing body weight.
**Adulthood**						
	Rivellese [107]	1997	16 ♂ & ♀ (NIDDM patients with hypertriglyeridemia)	56.0 ± 3.0	First two months: 0.96gr EPA and 1.59 g DHA per day Last four months: 0.64 gr EPA and 1.06 gr DHA per day	DHA and EPA significantly reduced plasma triglycerides and VLDL- triglycerides without significant changes in blood glucose control.
	Mori [105]	1999	56 ♂(overweight & hyperlipidemic)	49.1 ± 1.2	4 g/day DHA, EPA or olive oil (placebo) for 6 weeks	Purified DHA but not EPA reduced ambulatory BP and HR in mildly hyperlipidemic men.
	Mori [104]	2000	59 ♂ (overweight & hyperlipidemic)	50.6 ± 1.4	4 g/day DHA, EPA or olive oil (placebo) for 6 weeks	DHA enhances vasodilator mechanisms and attenuates constrictor responses in the forearm microcirculation.
	Woodman [102]	2003	♂ & ♀ (Hypertensive and diabetic)	40–75	4 g/day DHA, EPA or olive oil (placebo) for 6 weeks	DHA increased low density lipoprotein particle size
	Kelley [106]	2007	34 ♂	55.0 ± 2.0	7.5 g DHA-oil for 90 days	DHA supplementation for 45 d significantly decreased concentrations of fasting triacylglycerol, large VLDL, and intermediate-density lipoproteins and the mean diameter of VLDL particles.
	Sneddon [108]	2008	69 ♂	32.4 ± 2.3	3 g/day CLA + 3 g/day omega-3 LC-PUFAs	Supplementation with conjugated linoleic acids (CLAs) plus omega-3 LC-PUFAs prevents increased abdominal fat mass and raises fat-free mass and adiponectin levels in obese adults
	Micallef [6]	2009	124 ♂ & ♀	43.79 ± 2.22	Plasma levels of DHA & EPA	BMI, waist circumference and hip circumference were inversely correlated with *n*-3 PUFA, EPA and DHA (*p* < 0.05 for all) in the obese group. Obese individuals had significantly lower plasma concentrations of total *n*-3 PUFA, compared with healthy-weight individuals.
	Stirban [98]	2010	34 ♂ & ♀ (T2DM)	56.8 ± 8.3	2 g/d EPA & DHA for 6 weeks	Six weeks of supplementation with LC-PUFAs reduced the postprandial decrease in macrovascular function relative to placebo. LC-PUFAs supplementation improved postprandial microvascular function.
	Itariu [109]	2012	55 ♂ & ♀ (obese)	39.0 ± 2.0	3,36 g/d EPA & DHA for 8 weeks	*n*-3 PUFAs, which was well tolerated, decreased the gene expression of most analyzed inflammatory genes in subcutaneous adipose tissue (*p* <0.05) and increased production of anti-inflammatory eicosanoids in visceral adipose tissue and subcutaneous adipose tissue (*p* <0.05).
	Labonte [96]	2013	12 ♂ (obese +T2DM)	54.1 ± 7.2	3 g/d EPA & DHA for 8 weeks	In obese patients with T2DM, EPA&DHA supplementation did not affect the gene expression of pro-inflammatory cytokines in duodenal cells.
	Singhal [7]	2013	328 ♂ & ♀	28.1 ± 4.8	1.6 g/day DHA	DHA supplementation did not improve endothelial function in healthy adolescents. Only triglyceride and very low-density lipoprotein concentrations were significant lower in DHA-supplemented individuals compared with controls.
	McDonald [101]	2013	22 ♂ & ♀ Hypertensive and T2DM	58.6 ± 8.8	Daily supplementation of 1.8 g EPA and 1.5 g DHA for 8 weeks	LC-PUFAs diminish platelet superoxide production in T2DM hypertensive patients *in vivo*.
	Virtanen [99]	2013	2122 ♂	53.1 ± 5.1	Serum levels DHA, EPA, DPA	Men with higher serum level of EPA+DHA+DPA had a 33% lower multivariate-adjusted risk for T2DM. (Trend: *p* = 0.01)
**Mature and late adulthood**						
	Woodman [100]	2003	51 (39-♂ & 12-♀) (Hypertensive and diabetic)	61.2 ± 1.2	4 g/day DHA, EPA or olive oil (placebo) for 6 weeks	DHA supplementation significantly reduced collagen aggregation and collagen-stimulated thromboxane release.
	Lemaitre [122]	2003	54: Ischemic heart disease 125:non-fatal myocardial infarction 179: matched controls ♂ & ♀	79.1 ± 7.5	DHA & EPA plasma phospholipids	Higher combined dietary intake of DHA and EPA, and possibly α-linolenic acid, may lower the risk of fatal ischemic heart disease in older adults.
	Tsitouras [123]	2008	12 ♂ & ♀	66.1 ± 4.5	Supplemented with 4 g/day EPA and DHA	Insulin sensitivity increased significantly after 8 weeks on the EPA- and DHA-diet, and serum C-reactive protein was significantly reduced.
	Heine-Böring [5]	2010	1570 (686-♂ & 884-♀)	64.0 ± 5.42 - ♂ 64.0 ± 5.6 -♀	Food intake questionnaire; Dutch food composition table (DHA & EPA levels)	Subjects with a fish intake >19 g/d had a significantly lower prevalence of mild/moderate calcification. EPA plus DHA intake showed no significant associations.
	Djousse [48]	2011	3088 ♂ & ♀	75.0	Plasma phospholipids DHA and EPA	DHA is not associated with a higher incidence of T2DM, and individuals with higher EPA and DHA plasma concentrations had lower risk on T2DM.

DHA: docosahexaenoic acid; EPA: eicosapentaenoic acid; DPA: docosapentaenoic acid; CLA: conjugated linoleic acids; CHF: congestive heart failure; T2DM: diabetes mellitus type 2; NIDDM: non-insulin-dependent diabetes mellitus; VLDL: very-low-density-lipoprotein; N: number of participants; Age is represented in years and in Mean ± SD. Mean age >4 and ≤12 Year → childhood; Mean age >12 and ≤21 → adolescence; Mean age >21 and ≤60 → adulthood; Mean age >60 → middle and late adulthood.

### 2.5. DHA and Metabolic Diseases in Mature and Late Adulthood

Prevalence of metabolic diseases increases with age. Manzato* et al.*, for example, described that in Northern Italy metabolic diseases are present in 25.6% of elder men and in 48.1% of elder women [124]. Moreover, their prevalence was mainly due to high blood pressure (93% in both sexes) and to abdominal obesity (in 73% of women) [124]. The prevalence of cardiovascular diseases was significantly higher among subjects diagnosed with metabolic diseases [124]. Moreover, Czernichow* et al.* suggested that BMI was inversely correlated with resting capillary density, which suggests a lower baseline tissue perfusion; an association with higher vasomotor tone in overweight in the elderly [125].

The incidence of T2DM in older individuals was not associated with DHA plasma phospholipid levels [48] (Table 1). However, Tsitouras and colleagues reported that following a DHA and EPA diet for a period of eight weeks significantly increased insulin sensitivity in the elderly [123]. Omega-3 LC-PUFA consumption can provide protection against cardiovascular disease by improving vasoactivity, and decreasing blood pressure and inflammation. It was shown that a higher plasma concentration of DHA, EPA and DPA in older adults is associated with lower systolic blood pressure, pulse pressure and congestive heart failure (CHF), but not with diastolic blood pressure [50,126]. In addition, a higher combined DHA and EPA plasma phospholipids level was associated with a lower risk of fatal ischemic heart disease [122]. DHA supplementation significantly reduced collagen aggregation and collagen-stimulated thromboxane release, which indicates that highly purified DHA may be a more effective anti-thrombotic agent than EPA [100]. Furthermore, it was reported that healthy elderly people with a higher fish intake had a significantly lower prevalence of moderate coronary calcification [5]. On the other hand, intake of EPA plus DHA showed no significant associations [5]. These findings indicate that not only DHA is associated with a reduction of cardiovascular risk factors. It is a combination of these omega-3 fatty acids, DHA, EPA and DPA that seems to provide these beneficial effects on vascular health.

## 3. Conclusions

Omega-3 LC-PUFA supplementation in children and adolescents showed beneficial effects on reducing pathological characteristics in obesity, T2DM and CVD by decreasing adipocyte differentiation, increasing adipocyte apoptosis, promoting lipolysis, improving endothelial function, lowering blood pressure, decreasing inflammation, improving glucose tolerance and decreasing BMI [2,3,92,127,128]. Moreover, omega-3 LC-PUFAs reduce obesity symptoms and improve vascular health in adults and the elderly. Nevertheless, the impact of DHA can be ambiguous, and is affected by differences in age, gender, and health. Other limitations are found in differences in DHA dosing; concentrations differ between human studies, which makes comparison of research findings difficult.

DHA can spontaneously be converted via non-enzymatic oxidation by reactive oxygen species into neuroprostanes, isoprostanes, isofuranes, alkenes, alkanes and aldehydes (Figure 1). These lipid peroxides, previously thought to have a harmful effect on cells, can activate gene expression changes via peroxisome proliferator-activated receptors (PPARs) and nuclear factor-like 2 (Nfr2). In addition, these molecules can also have a direct influence on ion-channel conductance, activity of proteins/enzymes via carbonyl modification, inhibition of adhesion and atherosclerosis progression [129,130,131,132,133,134]. These recent findings emphasize a need for multi-nutrient diets, for example LC-PUFAs combined with vitamins and anti-oxidants. These dietary factors could inhibit or stimulate the molecular processes as described and, therefore, reinforce each other’s beneficial effects in an optimal combination. So, future research should focus on determining this optimal DHA-dose, depending on age and gender, and, secondly, combinations of several dietary factors like LC-PUFAs and anti-oxidants should be examined. In addition, a multi-nutrient diet could establish a multifactorial approach on inflammation and vasodilation, for instance.

DHA has been suggested to play a role in the prevention and better management of obesity, T2DM and cardiovascular disease. Recently, several studies have revealed that obesity, T2DM and CVD are correlated with the risk of dementia and AD in later life [135,136,137,138,139]. Therefore, DHA may protect against cognitive decline via two pathways; firstly, by reducing the development of diseases like obesity, T2DM and CVD. Secondly, by protecting against cognitive decline via maintaining membrane fluidity, increasing synaptogenesis, stimulating neurogenesis, decreasing neuroinflammation and improving cerebral blood flow [54,62,75,83,84,98,110,111,112,113,114,115,116,117,118,119,120]. Metabolic diseases like obesity, CVD and T2DM significantly and independently increase the risk of neurodegeneration and are therefore risk factors in the development of dementia and AD [140,141,142].

Further understanding is needed to define optimal doses per age and sex, in order to target these different metabolic diseases and to assess the relative efficiency of omega-3 LC-PUFAs, specifically DHA. Taken altogether, DHA could be one of the dietary protectors throughout a lifetime.
